# Supplementary material for: Mapping the evolution of fertility support policies in China: A content and instrumental analysis
Source: PLoS One. 2025 Oct 9;20(10):e0332137. doi: 10.1371/journal.pone.0332137 (PMC12510515; doi:10.1371/journal.pone.0332137)
Supplement: S1 Appendix — (ZIP) [file pone.0332137.s001.zip › S1 Appendix. 226 original policy documents/98-女职工劳动保护规定.doc]

女职工劳动保护规定

**发布部门：** [国务院](https://www.pkulaw.com/law/chl?Aggs.IssueDepartment=5) [C:\email\index](../../../../C:/email/index)

**发文字号：**中华人民共和国国务院令第9号

**发布日期：**1988.07.21

**实施日期：**1988.09.01

**时效性：** [失效](https://www.pkulaw.com/law/chl?Aggs.TimelinessDic=02)

**效力级别：** [行政法规](https://www.pkulaw.com/law/chl?Aggs.EffectivenessDic=XC0201)

**法规类别：** [劳动安全与劳动保护](https://www.pkulaw.com/law/chl?Aggs.Category=08706)

**失效依据：**[女职工劳动保护特别规定](javascript:TMP(173287,0,'chl'))

中华人民共和国国务院令
（第９号）

　　《女职工劳动保护规定》已经１９８８年６月２８日国务院第十一次常务会议通过，现予发布，自１９８８年９月１日起施行。

　　　　　　　　　　　　　　　　　　　　　　　　　　　总理　李鹏
1988年7月21日

女职工劳动保护规定

　　第一条　为维护女职工的合法权益，减少和解决女职工在劳动和工作（以下统称劳动）中因生理特点造成的特殊困难，保护其健康，以利于社会主义现代化建设，制定本规定。

　　第二条　本规定适用于中华人民共和国境内一切国家机关、人民团体、企业、事业单位（以下统称单位）的女职工。

　　第三条　凡适合妇女从事劳动的单位，不得拒绝招收女职工。

　　第四条　不得在女职工怀孕期、产期、哺乳期降低其基本工资，或者解除劳动合同。

　　第五条　禁止安排女职工从事矿山井下、国家规定的第四级体力劳动强度的劳动和其他女职工禁忌从事的劳动。

　　第六条　女职工在月经期间，所在单位不得安排其从事高空、低温、冷水和国家规定的第三级体力劳动强度的劳动。

　　第七条　女职工在怀孕期间，所在单位不得安排其从事国家规定的第三级体力劳动强度的劳动和孕期禁忌从事的劳动，不得在正常劳动日以外延长劳动时间；对不能胜任原劳动的，应当根据医务部门的证明，予以减轻劳动量或者安排其他劳动。

　　怀孕七个月以上（含七个月）的女职工，一般不得安排其从事夜班劳动；在劳动时间内应当安排一定的休息时间。

　　怀孕的女职工，在劳动时间内进行产前检查，应当算作劳动时间。

　　第八条　女职工产假为九十天，其中产前休假十五天。难产的，增加产假十五天。多胞胎生育的，每多生育一个婴儿，增加产假十五天。

　　女职工怀孕流产的，其所在单位应当根据医务部门的证明，给予一定时间的产假。

　　第九条　有不满一周岁婴儿的女职工，其所在单位应当在每班劳动时间内给予其两次哺乳（含人工喂养）时间，每次三十分钟。多胞胎生育的，每多哺乳一个婴儿，每次哺乳时间增加三十分钟。女职工每班劳动时间内的两次哺乳时间，可以合并使用。哺乳时间和在本单位内哺乳往返途中的时间，算作劳动时间。

　　第十条　女职工在哺乳期内，所在单位不得安排其从事国家规定的第三级体力劳动强度和哺乳期禁忌从事的劳动，不得延长其劳动时间，一般不得安排其从事夜班劳动。

　　第十一条　女职工比较多的单位应当按照国家有关规定，以自办或者联办的形式，逐步建立女职工卫生室、孕妇休息室、哺乳室、托儿所、幼儿园等设施，并妥善解决女职工在生理卫生、哺乳、照料婴儿方面的困难。

　　第十二条　女职工劳动保护的权益受到侵害时，有权向所在单位的主管部门或者当地劳动部门提出申诉。受理申诉的部门应当自收到申诉书之日起三十日内作出处理决定；女职工对处理决定不服的，可以在收到处理决定书之日起十五日内向人民法院起诉。

　　第十三条　对违反本规定侵害女职工劳动保护权益的单位负责人及其直接责任人员，其所在单位的主管部门，应当根据情节轻重，给予行政处分，并责令该单位给予被侵害女职工合理的经济补偿；构成犯罪的，由司法机关依法追究刑事责任。

　　第十四条　各级劳动部门负责对本规定的执行进行检查。

　　各级卫生部门和工会、妇联组织有权对本规定的执行进行监督。

　　第十五条　女职工违反国家有关计划生育规定的，其劳动保护应当按照国家有关计划生育规定办理，不适用本规定。

　　第十六条　女职工因生理特点禁忌从事劳动的范围由劳动部规定。

　　第十七条　省、自治区、直辖市人民政府可以根据本规定，制定具体办法。

　　第十八条　本规定由劳动部负责解释。

　　第十九条　本规定自１９８８年９月１日起施行。１９５３年１月２日政务院修正发布的《[中华人民共和国劳动保险条例](https://www.pkulaw.com/chl/96be87573ffd9605bdfb.html)》中有关女工人、女职员生育待遇的规定和１９５５年４月２６日《[国务院关于女工作人员生产假期的通知](https://www.pkulaw.com/chl/1209817908083dddbdfb.html)》同时废止。
